# Effects of Daytime Dry Fasting on Hydration, Glucose Metabolism and Circadian Phase: A Prospective Exploratory Cohort Study in Bahá'í Volunteers

**DOI:** 10.3389/fnut.2021.662310

**Published:** 2021-07-29

**Authors:** Daniela A. Koppold-Liebscher, Caroline Klatte, Sarah Demmrich, Julia Schwarz, Farid I. Kandil, Nico Steckhan, Raphaela Ring, Christian S. Kessler, Michael Jeitler, Barbara Koller, Bharath Ananthasubramaniam, Clemens Eisenmann, Anja Mähler, Michael Boschmann, Achim Kramer, Andreas Michalsen

**Affiliations:** ^1^Institute of Social Medicine, Epidemiology and Health Economics, Charité—Universitätsmedizin Berlin, Corporate Member of Freie Universität Berlin, Humboldt-Universität zu Berlin, and Berlin Institute of Health, Berlin, Germany; ^2^Department of Internal and Integrative Medicine, Immanuel Hospital Berlin, Berlin, Germany; ^3^Department of Sociology, Cluster of Excellence Religion and Politics, University of Münster, Münster, Germany; ^4^Department of Oecotrophology, Hochschule Niederrhein, University of Applied Science, Mönchengladbach, Germany; ^5^Department of Neurology, Campus Benjamin Franklin, Charité Universitätsmedizin Berlin, Berlin, Germany; ^6^Connected Healthcare, Hasso Plattner Institute, University of Potsdam, Potsdam, Germany; ^7^Laboratory of Chronobiology, Institute of Medical Immunology, Charité—Universitätsmedizin Berlin, Corporate Member of Freie Universität Berlin, Humboldt-Universität zu Berlin, and Berlin Institute of Health, Berlin, Germany; ^8^Institute for Theoretical Biology, Charité Universitätsmedizin Berlin, Berlin, Germany; ^9^Department of Sociology, University of Konstanz, Konstanz, Germany; ^10^Experimental and Clinical Research Center—a Joint Cooperation Between Charité-Universitätsmedizin Berlin and Max Delbrueck Center for Molecular Medicine, Berlin, Germany; ^11^NeuroCure Clinical Research Center, Charité-Universitätsmedizin Berlin, Berlin, Germany

**Keywords:** hydration, religious, intermittent fasting, chronobiology, water deprivation, time-restricted eating, fasting, diurnal fasting

## Abstract

**Background:** Religiously motivated Bahá'í fasting (BF) is a form of intermittent dry fasting celebrated by abstaining from food and drinks during daylight hours every year in March for 19 consecutive days.

**Aim:** To test the safety and effects of BF on hydration, metabolism, and the circadian clock.

**Methods:** Thirty-four healthy Bahá'í volunteers (15 women) participated in this prospective, exploratory cohort study. Laboratory examinations were carried out in four study visits: before fasting (V0), in the third week of fasting (V1) as well as 3 weeks (V3) and 3 months (V4) after fasting. Data collection included blood and urine samples, anthropometric measurements and bioelectrical impedance analysis. At V0 and V1, 24- and 12-hour urine and serum osmolality were measured. At V0–V2, alterations in the circadian clock phase were monitored in 16 participants. Our study was augmented by an additional survey with 144 healthy Bahá'í volunteers filling out questionnaires and with subgroups attending metabolic measurements (*n* = 11) and qualitative interviews (*n* = 13), the results of which will be published separately.

**Results:** Exploratory data analysis revealed that serum osmolality (*n* = 34, *p* < 0.001) and 24-hour urine osmolality (n = 34, *p* = 0.003) decreased during daytime fasting but remained largely within the physiological range and returned to pre-fasting levels during night hours. BMI (body mass index), total body fat mass, and resting metabolic rate decreased during fasting (n = 34, *p* < 0.001), while body cell mass and body water appeared unchanged. The circadian phase estimated by transcript biomarkers of blood monocytes advanced by 1.1 h (*n* = 16, *p* < 0.005) during fasting and returned to pre-fasting values 3 weeks after fasting. Most observed changes were not detectable anymore 3 months after fasting.

**Conclusions:** Results indicate that BF (Bahá'í fasting) is safe, has no negative effects on hydration, can improve fat metabolism and can cause transient phase shifts of circadian rhythms.

**Trial Registration:**https://www.clinicaltrials.gov/, identifier: NCT03443739.

## Introduction

Fasting with its preventive and therapeutic effects has, in general, been explored more and more over the past decade (1–10). However, dry fasting (DF) has not received as much attention in the scientific community. Most studies in this regard have focused on the Ramadan fast (11, 12), not specifically exploring DF as such, while serious consequences of a dysregulated body fluid balance are clear for every clinician (13), making studies on DF difficult to conduct.

However, Ramadan fasting is a complex model for DF, as the daily duration of fasting varies greatly over the years (from 11 to 22 hours a day, depending on the season and geographic location) (14). Apart from studies on Ramadan fasting, only a few studies have focused on DF itself (15–20). From these, only Papagiannopoulos et al. and Papagiannopoulos-Vatopaidinos et al. (15, 20) have focused on the physiology of prolonged DF, examining 10 participants each for 5 consecutive days of water and food deprivation.

The Bahá'í religion is monotheistic and was founded by Bahá'u'lláh (1817–1892) (21). Fasting is seen by Bahá'ís as one of the most significant spiritual duties of a healthy individual (22). The sick, the elderly, as well as children under 15 years of age and pregnant, menstruating, or nursing women are exempt from the religious duty to fast (22). Worldwide, the followers of the Bahá'í religion fast every year in March, abstaining from food and drink from sunrise to sunset for 19 consecutive days (22). This practice could best be described as an intermittent dry fast. No additional dietary regulations exist for believers during the fasting period or in general, except that consumption of alcoholic beverages is prohibited throughout the year. In contrast to Ramadan fasting, Bahá'í fasting (BF) has a fixed date in the year, is of shorter duration (i.e. 19 days) and, since it takes place in March, fasting time varies only between 10–13 hours daily. At the study site in Berlin/Germany, the exact duration is 10.90 hours at the beginning and 12.12 hours at the end of the fasting period. This makes it a good model for exploring the effects of intermittent dry fasting (IDF) on healthy adults, as no relevant changes occur in climate or timing when comparing data from different years or places. Since research has suggested that Ramadan fasting causes alterations of normal circadian rhythms (23), we also explored the effects of BF on the circadian phase (chronotype).

Thus, in this exploratory study, fluid balance in healthy adults during a religiously motivated IDF was assessed. Additionally, it was explored, whether known physiological mechanisms of fasting are also activated through this kind of fasting, which is shorter in duration than other fasting intervals of, for example, time-restricted eating (1) or intermittent fasting (5). More specifically, the aim was to assess urine and serum osmolality, renal and liver function, blood fatty acids, ketones, blood gas analysis, and anthropometric variables, as well as effects on normal daily patterns of eating and drinking. The collected data should serve to generate hypotheses for future studies in the field of IDF in general as well as assessing the safety and effects of BF.

## Methods

### Study Design

This longitudinal, exploratory cohort study focussed on changes in physiological parameters. It was augmented by a mixed-methods study on Bahá'í fasting.

This study represents a quasi-experiment with a longitudinal observational design (24). As fasting time and duration are fixed for all Bahá'ís by religious provisions, there was no possibility to randomize the volunteers into a fasting and a non- (or delayed-) fasting group. Using a control group with a non-religious motivation could lead to a bias, as religiously motivated people may have higher compliance rates to dietary norms as well as engage in different health-related behaviors compared with non-adherents (25). The external laboratory personnel, as well as the statistician, were blinded. All other study personnel were involved directly with the study participants during visits. Therefore, no blinding was possible in this case.

The study protocol was approved by the institutional review board of Charité Universitätsmedizin Berlin (Charitéplatz 1, 10117 Berlin) in January 2018 (ID: EA4/216/17), was registered with ClinicalTrials.gov (ID: NCT03443739), and carried out according to the standards of the Declaration of Helsinki. Written informed consent was obtained from all the participants prior to study entry.

### Setting

**Figure 1:**
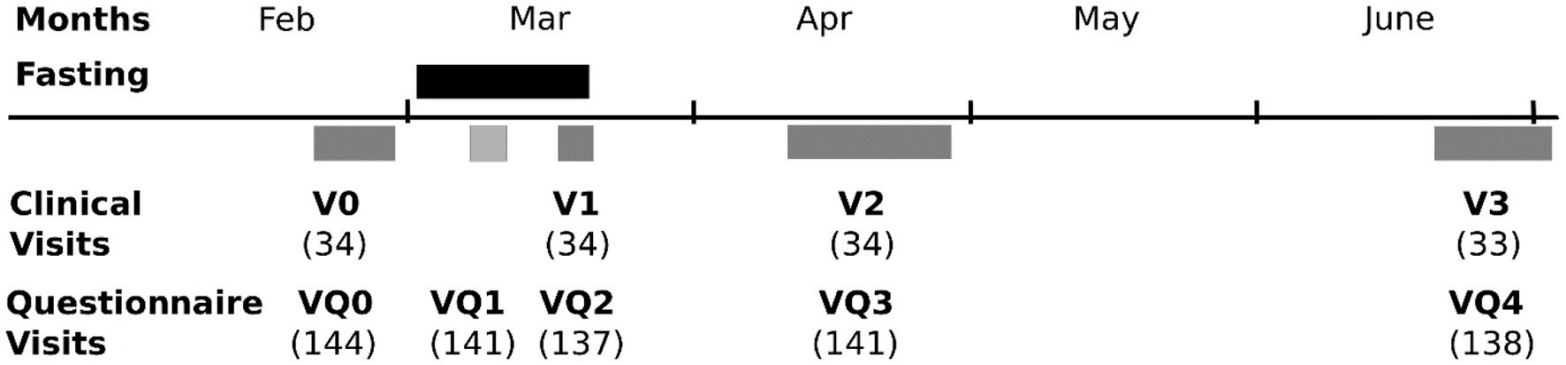
A flow chart of visits with participant numbers.

Three departments of the same university (Charité Universitätsmedizin Berlin) cooperated for this study: the Department of Integrative Medicine at the Institute of Social Medicine, Epidemiology and Health Economics (IM), the Experimental and Clinical Research Center (ECRC), and the Department of Chronobiology (DC). The two latter departments conducted sub-studies. The main study site was at the Department of Integrative Medicine. Physiological data comprising urine and blood samples, as well as anthropometric measurements, were acquired at the four in-house visits in the Department of Integrative Medicine at the Institute of Social Medicine, Epidemiology and Health Economics (IM) or, for a subgroup, at the Experimental and Clinical Research Center (ECRC). The visits were conducted in the 2 weeks before the beginning of the fasting period (V0), in the last week of the fast (V1), 3 weeks after the end of the fast (V2), and finally, 3 months after the end of the fasting period (V3). Figure 1 shows these visits along with the additional other visits for the psychometric questionnaires of the companion study and the other sub-studies.

### Participants

Original first recruitment was conducted *via* the National Spiritual Assembly of the Bahá'ís of Germany (23), who spread the information via email to all Bahá'ís in Germany. For the aims of this study, 172 healthy volunteers were screened, and 144 were considered eligible (see below) for the questionnaire survey and were enclosed between January and February 2018. All eligible individuals filled out electronic questionnaires on subjective physical and psychological effects of Bahá'í fasting (group “BQ”).

Of these, all the participants living in the wider region of Berlin were invited to participate in the additional laboratory tests reported upon here. Thirty-four subjects could be enclosed in this sub-study with its tests for physiological parameters (group “PP”), giving their informed written consent. Again, 17 of these 34 participants agreed to donate additional blood samples for chronobiological measurements and to answer additional questionnaires (group “CB”). The results of the psychometric questionnaires, as well as those of individual in-depth and focus group interviews and another subgroup concentrating on metabolic responses, will be reported separately.

#### Eligibility Criteria

The participants were screened at the IM and then referred to all sub-studies.

Inclusion criteria were registered membership in the Bahá'í community, planned adherence to fasting in the upcoming fasting period and age between 18 and 69 years (children and elderly are exempt from the religious duty to fast, and, because of the necessity for informed consent, no youth between 15 and 17 years were recruited for the study).

Exclusion criteria were: scheduled interruption of the fast for more than 5 days (for e.g., due to planned travel), pregnancy or breastfeeding, serious physical, or psychological illness, known eating disorder, participation in another study, no email address (because of electronic questionnaires). Individuals who worked in shifts and planned long-distance travel shortly before and during the study period were also excluded from eligibility for the chronobiological measurements.

### Outcomes/Variables

Primary outcome measures were the hydration status of fasting individuals and changes in serum osmolality and urine osmolality in 12- and 24-hour-urine samples. Secondary outcome measures were anthropometric parameters, such as body weight, BMI, body composition via bioelectrical impedance analysis (BIA, measured by the octapolar BIACORPUS RX 4004M®) and the waist-to-hip ratio. The resting metabolic rate was calculated by an algorithm implemented in the BIA software provided by the manufacturer. Blood pressure, heart rate, and standard blood count were assessed alongside metabolic parameters, such as ketone bodies in capillary blood (on the spot, with ACCU-CHEK® device) and urine, blood glucose levels, glycosylated hemoglobin (HbA1c), fructosamine, C-reactive protein, liver enzymes, and serum cholesterol. Additionally, the fluid balance was assessed by kidney parameters, 12- and 24-hour urine creatinine clearance, serum electrolytes, acid-based balance measured by blood gas analysis (on the spot, with the ABL80 FLEX® blood gas analyser), 24-hour and spontaneous urine osmolality, 24-hour urine specific gravity, and cystatin C. All laboratory parameters were measured at Labor Berlin, unless otherwise indicated. As most laboratory measurements had to be carried out in a fasting state, they were carried out between 7:30 a.m. and 11:00 a.m. outside of the fasting period, while, during BF, as most subjects would have breakfast before sunrise, the measurements were done between 4:30 p.m. and 6:30 p.m., if not otherwise indicated.

In group CB (17 subjects), an additional blood sample was taken between ~8 a.m. and ~10 a.m. at V0, V1, and V2 to assess the circadian phase (chronotype) (Supplementary Table 1) (26). Note that the determination of the circadian phase with the BodyTime assay is independent of the time of sample collection (26). Briefly, monocytes were sorted from whole blood, using magnetic cell sorting, total RNA was prepared, and the expression of 24 biomarker genes was analyzed, using NanoString technology. Based on these data, the body time algorithm allowed the prediction of the circadian phase. In this subgroup, the validated Munich Chronotype questionnaire (27) was also used.

Timing of the measurements constituted a challenge. Due to capacity constraints of the study centers, measurements could not all be done at the same time. Therefore, the measurements had to be spread over the last week of BF and start 2 hours before breaking the fast for dinner so that the mean fasting time for our measurements was 17 fasting days and 11 hours of daily fasting duration.

For a better overview of the whole study setting, we also mention the following measurements, the results of which will be published separately: Body composition was measured by air-displacement plethysmography, parameters of systemic energy metabolism by indirect calorimetry, and parameters of adipose tissue and skeletal muscle metabolism by microdialysis in two subgroups. Furthermore, validated questionnaires on quality of life, mood, mindfulness, and spirituality were used in electronic form for the BQ group. Additionally, the focus group and individual interviews were carried out by a trained sociologist.

### Bias

Recruitment for both this study and the accompanying ones was based on voluntary participation and may thus have introduced a sampling bias in favor of motivated and health-conscious subjects. Apart from that, the implementation of an intervention-group-only design is, in so far, justified as the aim of the study was to explore the safety and general health effects of BF, using pre- and post-fasting observations to explore the strength and duration of the fasting effects.

### Study Size

The study was planned as an exploratory study to help generate hypotheses for future studies. All *p*-values lower than 0.10 are regarded as potentially interesting and those below 0.05 as strongly interesting for future studies. The number of the participants (*n* = 34) allows to detect all high-sized and medium-sized effects of *f* ≥ 0.16, corresponding to a Cohen's *d* ≥ 0.32 (28) under the assumption of standard parameters [alpha = 0.05, beta = 0.20 (power of 80%)] and a higher correlation between measures of 0.7 (based on previous in-house data).

### Statistical Methods

As blood parameters are typically not normally distributed, all analyses were conducted, using non-parametric tests. The Friedman test, a counterpart to a one-factorial repeated measures ANOVA, was used to compare the physiological parameters across the four (three) clinical visits, while pairwise *post-hoc* analyses were applied, using the Wilcoxon's signed-rank test. As usual, in exploratory studies, resulting *p*-values are presented as such and not compared against any adjusted alpha value.

#### Missing Data

Some physiological data were missing during the laboratory failures and one subject not attending the last visit. The Little test indicated that the missing data were completely at random (“MCAR”) (29) and were replaced using multiple imputation methods (“multiple imputer”) implemented in the Python's (v. 3.7) SciPy library.

## Results

### Participants

Thirty-four volunteers (15 w, 19 m, age: 41.09 ± 14.54 years) practicing BF and living in or near Berlin participated in the visits and tests of physiological parameters (group “PP”). They had a mean BMI of 25.74 ± 5.13 kg/m^2^, corresponding well to the average BMI of 26 kg/m^2^ in Germany (30). Further sociodemographic data and baseline characteristics of these participants are shown in Tables 1, 2, respectively. Confidence intervals of the baseline laboratory parameters are clinically unremarkable.

**Table 1:** Sociodemographic characteristics of the participants in the laboratory measurements.

		**Option 1**		**Option 2**		**Option 3**		**Option 4**		**Option 5**	
	***n***	**Answer**	***n* (%)**	**Answer**	***n* (%)**	**Answer**	***n* (%)**	**Answer**	***n* (%)**	**Answer**	***n* (%)**
Gender	34	Female	15 (44.1)	Male	19 (55.9)						
Graduation	33	A-level	10 (30.3)	University	22 (66.7)	Other	1 (3)				
Gross income per year [kEUR]	33	<20	18 (54.5)	20–40	4 (12.1)	40–60	5 (15.2)	60–80	2 (6.1)	>80	4 (12.1)
Job	33	Self-Employed	7 (21.2)	Employee	11 (33.3)	Worker	2 (6.1)	Student	10 (30.3)	Other	3 (9.1)

**Table 2:** Characteristics of participants at baseline (V0).

	**Original data**				**Multiply imputed data**			
	**N**	**Mean ± SD**	**Median (IQR)**	**95% CI**	**N**	**Mean ± SD**	**Median (IQR)**	**95% CI**
Age	34	41.09 ± 14.54	39.5 (29)	36.02; 46.16				
Hight, cm	34	173.49 ± 9.12	175.5 (15.1)	170.31; 176.67				
Weight, kg	34	77.97 ± 19.19	75.3 (19.4)	71.28; 84.67				
BMI, kg/m^2^	34	25.74 ± 5.13	24.52 (6.36)	23.95; 27.53				
WHR^a^	34	0.90 ± 0.091	0.90 (0.13)	0.87; 0.93				
Blood pressure sys^b^, mmHg	33	123.94 ± 18.32	120 (25)	117.44; 130.43	34	124.25 ± 18.13	120 (25)	117.93; 130.58
Blood pressure dia^c^, mmHg	33	79.09 ± 10.71	80 (10)	79.09; 82.89	34	79.4 ± 10.70	80 (10)	75.66; 83.13
ß-Hydroxybutyrate, mmol/l	32	0.17 ± 0.19	0.15 (0.3)	0.10; 0.24	34	0.18 ± 0.20	0.18 (0.3)	0.11; 0.25
Glucose, mg/dl	33	83.48 ± 8.16	84 (13)	80.59; 86.38	34	83.07 ± 8.4	84 (13)	80.1; 86
HbA1c, mmol/mol Hb	34	33.78 ± 3.80	33.3 (4.9)	32.45; 35.10				
Fructosamine, μmol/l	34	239.56 ± 20.49	243.5 (24)	232.41; 246.71				
Body fat, kg	34	22.968 ± 10.81	19.1 (14.3)	19.20; 26.74				
Body water, l	34	39.84 ± 9.56	38.7 (13.5)	36.51; 43.18				
Body cell mass, kg	34	28.83 ± 7.89	29.8 (10.25)	26.08; 31.59				
Resting metabolic rate, kcal	34	1,655.94 ± 323.87	1,626 (447)	1,542.94; 1,768.95				
Haematocrit, l/l	34	0.412 ± 0.033	0.42 (0.04)	0.40; 0.42				
Creatinine, mg/dl	33	0.79 ± 0.13	0.77 (0.21)	0.74; 0.83	34	0.79 ± 0.12	0.77 (0.21)	0.74; 0.83
Cystatin C, mg/l	33	0.911 ± 0.132	0.87 (0.18)	0.86; 0.96	34	0.92 ± 0.13	0.88 (0.2)	0.87; 0.96
GFR (CKD-EPI)	33	89.94 ± 3.58	91 (0)	88.67; 91.21	34	89.89 ± 3.53	91 (0)	88.66; 91.53
HDL, mg/dl	33	59.58 ± 15.68	58 (25)	54.02; 65.13	34	59.61 ± 14.44	58.5 (25)	54.22; 65.00
LDL, mg/dl	33	117.24 ± 27.90	119 (44)	107.35; 127.13	34	117.49 ± 27.51	119 (43)	107.89; 127.09
Sodium (plasma), mmol/l	33	141.33 ± 1.74	141 (32)	140.71; 141.95	34	141.81 ± 1.89	141 (32)	140.81; 142.13
Potassium (plasma), mmol/l	33	4.00 ± 0.29	4 (0.4)	3.90; 4.11	34	3.99 ± 0.31	4 (0.4)	3.88; 4.09
Sodium (bga), mmol/l	31	142.06 ± 1.57	142 (32)	141.49; 142.64	34	144.79 ± 20.03	142 (32)	137.8; 151.78
Potassium (bga), mmol/l	31	3.95 ± 0.23	4 (0.3)	3.86; 4.03	34	3.94 ± 2.08	4 (0.3)	3.22; 4.67
Osmolality plasma, mosmol/kg	33	289.76 ± 4	290 (5)	288.34; 291.18	34	289.61 ± 4.03	289.5 (4)	288.21; 291.02
Osmolality urine 24 h, mosmol/kg	33	471.36 ± 158.36	449 (235)	415.21; 527.52	34	532.16 ± 387.26	454 (257)	397.03; 667.28
Osmolality urine 12-h (6 a.m.−6 p.m.), mosm/kg	31	411 ± 161.98	389 (156)	351.59; 470.41	34	429.88 ± 206.54	399.5 (177)	357.82; 501.94
Osmolality urine 12-h (6 p.m.−6 a.m.), mosm/kg	33	562.73 ± 219.63	550 (347)	484.85; 640.60	34	566.33 ± 217.29	560 (336)	490.51; 642.14

a *WHR, waist-to-hip ratio;*

b *sys, systolic;*

c *dia, diastolic*.

Of these 34 volunteers, 17 subjects (the “CB” subgroup) donated additional blood samples for chronobiological measurements. One participant was not able to attend the last follow-up visit in person. While part of the visit could be conducted via telephone (questionnaires), no physiological samples could be obtained. These, as well as missing laboratory data from some subjects for some visits due to a laboratory failure, were filled in by multiple imputations (see Methods).

### Outcomes

#### Primary Outcome: Hydration Markers

**Figure 2:**
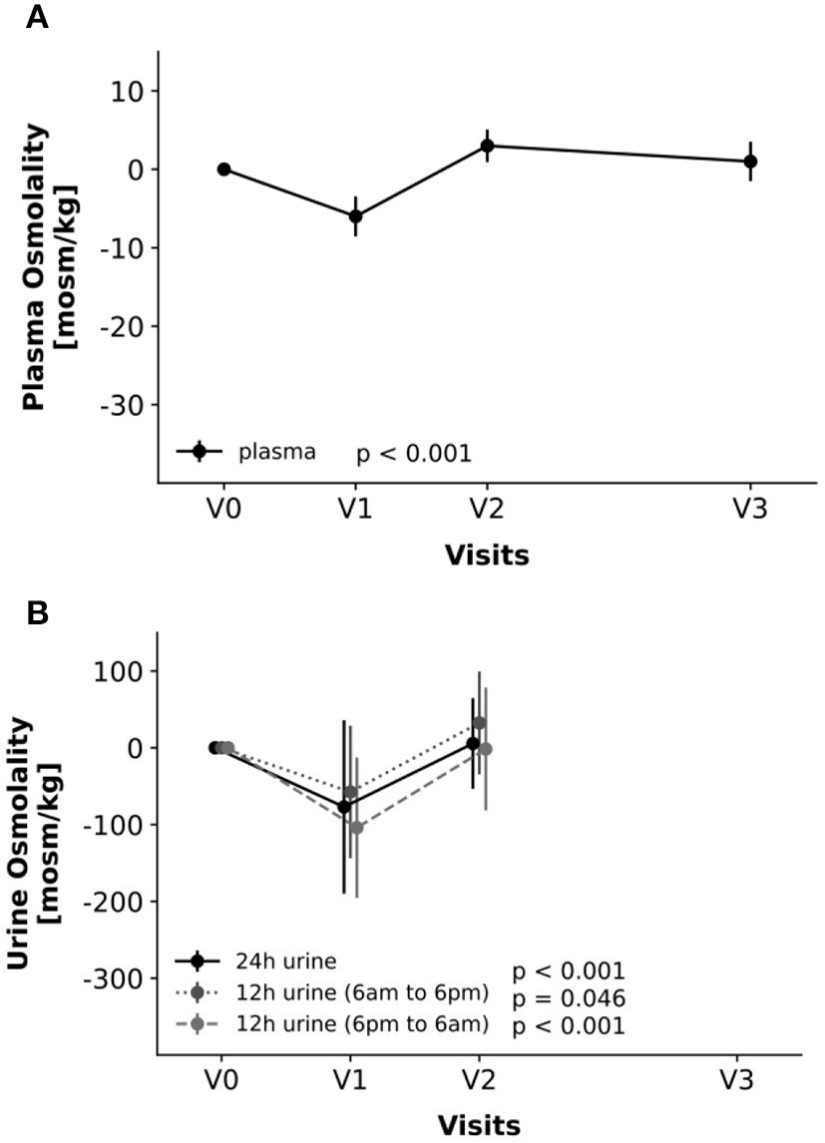
Changes in parameters of hydration, i.e., plasma osmolality **(A)** and urine osmolality in 24-h, 12-h from 6 a.m.−6 p.m. and 12-h from 6 p.m.−6 a.m. samples **(B)**. Changes relative to V0-values. Line graphs and error bars represent means and 95%-confidence intervals accordingly.

Plasma osmolality changed during fasting [χ^2^(3) = 19.61, *P* < 0.001]. It decreased from V0 to V1 and increased to baseline (V0) or even higher levels from V1 to V2, returning to baseline levels by V3 (Table 3, Figure 2A). However, all these changes were within the physiological range.

**Table 3:** Anthropometric, blood, and urine parameters before, during, and after 19 days of Bahá'í fasting (BF).

	**Reference** **range**	**V0**	**V1**	**V2**	**V3**	**Friedmann** **main analysis**		**V1 vs. V0**		**V1 vs. V2**		**V2 vs. V0**		**V3 vs. V0**		**V1 vs. V3**		**V2 vs. V3**	
						**χ^2^** **(3)**	***p***	***z***	***p***	***z***	***p***	***z***	***p***	***z***	***p***	***z***	***p***	***z***	***p***
Weight, kg		75.3 (19.4)	72.75 (21.8)	74.2 (20.9)	75.8 (18.8)	18.027	<0.001	4.180	<0.001	−2.114	0.207	2.067	0.233	1.644	0.601	−2.536	0.067	−0.423	1.000
BMI, kg/m^2^		24.52 (6.18)	24.24 (6.23)	24.52 (5.71)	24.43 (5.3)	17.74	<0.001	3.983	<0.001	2.359	<0.001	2.342	0.019	2.069	0.039	1.428	0.153	0.239	0.811
WHR		0.895 (0.13)	0.92 (0.13)	0.88 (0.09)	0.87 (0.14)	30.526	<0.001	0.892	1.000	−2.771	0.034	1.879	0.362	4.086	<0.001	−4.978	<0.001	−2.207	0.164
Body fat, kg		19.1 (13.48)	19.05 (12.7)	19.25 (12.98)	18.25 (13.55)	31.83	<0.001	4.36	<0.001	2.248	0.025	3.359	0.001	3.915	<0.001	0.53	0.596	1.103	0.27
Body water, l		38.7 (13.5)	38.95 (15.8)	38.5 (13.1)	39 (13.4)	9.107	0.028	0.282	1.000	−0.845	1.000	1.127	1.000	2.724	0.039	−2.442	0.088	−1.597	0.662
BCM, kg		29.8 (10.25)	29.7 (12.17)	29.83 (10.92)	29.7 (10)	13.823	0.006	1.409	0.953	−2.667	0.045	1.268	1.000	2.020	0.261	−3.429	0.004	−0.751	1.000
BMR, kcal/d		1,626 (422.25)	1,612 (410.0)	1,617 (404.75)	1,604 (383.25)	23.01	<0.001	4.496	<0.001	3.411	0.001	1.958	0.05	2.043	0.041	1.983	0.047	0.128	0.898
SBP, mmHg		120 (25)	120 (21)	115 (20)	120 (20)	12.229	0.007	1.503	0.797	−1.409	0.953	2.912	0.022	0.094	1.000	−1.409	0.953	−2.818	0.029
DBP, mmHg		80 (10)	70 (10)	70 (10)	75 (10)	13.246	0.004	2.959	0.019	0.000	1.000	2.959	0.019	2.160	0.184	−0.798	1.000	−0.798	1.000
Hematocrit	0.395–0.505	0.416 (0.036)	0.40 (0.05)	0.41 (0.03)	0.408 (0.042)	11.620	0.009	3.006	0.016	−2.818	0.029	0.188	1.000	1.127	1.000	−1.879	0.362	−0.939	1.000
Glucose, mg/dl	60–110	84.0 (12.5)	72.5 (7.75)	84.0 (9.75)	84.71 (13.25)	43.45	<0.001	4.59	<0.001	4.915	<0.001	0.658	0.51	1.325	0.185	4.958	<0.001	0.932	0.351
HbA1c, mmol/mol Hb	<42.0	33.3 (4.4)	32.75 (5.23)	31.65 (5.4)	33.16 (5.12)	48.92	<0.001	1.222	0.222	4.838	<0.001	4.471	<0.001	3.368	0.001	3.069	0.002	4.428	<0.001
Fructosamine, μmol/l	205–285	243.5 (24)	253 (32)	248 (22)	248 (17)	32.056	<0.001	5.401	<0.001	−2.630	0.051	2.771	0.034	4.039	<0.001	−1.362	1.000	−1.268	1.000
3-OH-B, mmol/l		0.18 (0.3)	0.15 (0.3)	0.1 (0.2)	0 (0.2)	5.000	0.172												
HDL, mg/dl	>45	58.5 (25)	58.5 (19)	58 (23)	56 (17)	10.495	0.015	0.094	1.000	−0.047	1.000	0.141	1.000	2.489	0.077	−2.583	0.059	−2.630	0.051
LDL, mg/dl	<130	119 (43)	115.5 (47)	117 (45)	119 (33)	2.063	0.559												
Creatinine, mg/dl	0.70–1.20	0.765 (0.21)	0.80 (0.19)	0.81 (0.24)	0.80 (0.21)	7.410	0.060												
Cystatin C, mg/l	0.47–1.09	0.88 (0.2)	0.89 (0.16)	0.9 (0.14)	0.92 (0.21)	6.960	0.073												
GFR (CKD-EPI)		91 (0)	91 (0)	91 (1)	91 (36)	1.943	0.584												
pNa^+^, mmol/l	136–145	141 (36)	142 (36)	142 (36)	141 (3)	3.884	0.274												
pK^+^, mmol/l	3.4–4.5	4.0 (0.4)	3.8 (0.4)	4.1 (0.3)	4.0 (0.4)	16.111	0.001	1.315	1.000	−3.851	0.001	2.536	0.067	0.282	1.000	−1.597	0.662	−2.254	0.145
cNa^+^, mmol/l	136–145	142 (36)	142 (36)	141.5 (3)	141 (3)	6.683	0.083												
cK^+^, mmol/l	3.3–5.1	4.0 (0.3)	3.8 (0.4)	4.1 (0.3)	4.0 (0.2)	10.867	0.012	1.221	1.000	−2.771	0.034	1.550	0.727	1.362	1.000	−2.583	0.059	−0.188	1.000
pOsmolality, mosmol/kg	280–300	289.5 (4.0)	284.0 (11.0)	291.5 (4.75)	289.0 (7.5)	18.6	<0.001	3.112	0.002	4.035	<0.001	2.06	0.039	0.402	0.688	2.821	0.005	2.000	0.045
uOsmolality, 24 h, mosmol/kg	50–1,400	454.0 (237.0)	380.0 (190.0)	448.06 (173.26)		13.91	<0.001	2.419	0.016	2.376	0.017	0.282	0.778						
uOsmolality, d−12 h, mosmol/kg	50–1,400	413.0 (195.5)	352.5 (162.75)	417.5 (196.36)		6.18	0.0456	1.658	0.097	2.676	0.007	1.265	0.206						
uOsmolality, *n*−12 h, mosmol/kg	50–1,400	560.0 (353.75)	406.0 (292.25)	524.5 (294.25)		14.51	<0.001	2.753	0.006	3.009	0.003	0.342	0.732						

*Data are given as the median and interquartile range (IQR). n = 34 for V0, V1, V2, V3; BMI, body mass index; BCM, body cell mass; RMR, resting metabolic rate; SBP/DBP, systolic/diastolic blood pressure; 3-OH-B, 3-Hydroxy-Butyrate; HDL, high-density lipoprotein; LDL, low-density lipoprotein; GFR, glomerular filtration rate; pNa^+^/pK^+^, plasma Na^+^/K^+^; cNa^+^/pK^+^, capillary Na^+^/K^+^; pOsmolality, plasma osmolality; uOsmolality, urine osmolality; d−12 h, urine sample 6:00 a.m.−6:00 p.m.; n−12 h, 6:00 p.m.−6:00 a.m.; V0: before BF; V1: during third week of BF; V2: 3 weeks after BF; V3: 3 months after BF*.

Osmolality of the 24-hour urine (Figure 2B) changed similarly [χ^2^(3) = 13.91, *P* < 0.001], with a decrease from V0 to V1 and a subsequent increase from V1 to V2 to baseline levels. In-depth analysis of the samples, however, revealed that this initial decrease was mainly due to a distinct decrease in nocturnal urine osmolality (sampled from 6 p.m. to 6 a.m.), whereas changes in diurnal urine were less marked. Overall, these changes were largely within the physiological range and, therefore, clinically not relevant.

#### Kidney Values and Electrolytes

The glomerular filtration rate, as estimated by the equation of the Chronic Kidney Disease Epidemiology Collaboration (GFR via CKD-EPI), creatinine, and cystatin C did not change relevantly during fasting (GFR: *P* = 0.584, creatinine: *p* = 0.060, and cystatin C: *p* = 0.073). Creatinine showed a tendency to increase slightly during fasting (V0–V1: 0.03 ± 0.09 mg/dl) and remained on this level after fasting when compared with baseline (V0–V2: 0.04 ± 0.08 mg/dl).

Sodium and potassium were measured in the plasma and by blood gas analysis (BGA). Sodium measures did not change relevantly, neither in plasma nor in the BGA. Potassium showed a slight decrease in both plasma [χ^2^(3) = 16.111, *p* = 0.001] and BGA measurements [χ^2^(3) = 10.867, *p* = 0.012]. *Post-hoc* testing shows that this result is mainly based on the increase between V1 and V2 to a possibly over-compensational level at V2 for both laboratory values.

#### Anthropometry

**Figure 3:**
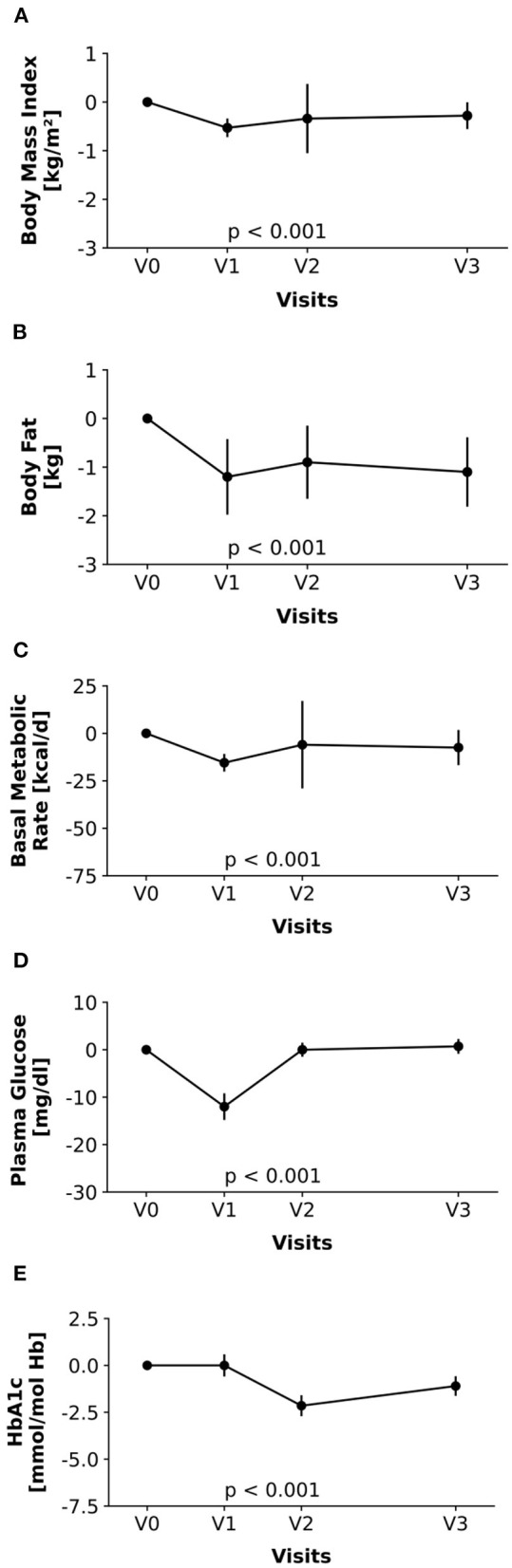
Changes in metabolic parameters over the study period (**A:** BMI, **B:** body fat mass, **C:** resting metabolic rate, **D:** glucose, and **E:** HbA1c). Changes relative to V0-values. Line graphs and error bars represent means and 95%-confidence intervals accordingly.

Body weight [χ^2^(3) = 18.027, *p* < 0.001], BMI [χ^2^(3) = 18,027, *p* < 0.001], WHR [χ^2^(3) = 30.526, *p* < 0.001], body fat [χ^2^(3) = 30.154, *p* < 0.001], body cell mass [χ^2^(3) = 13.823, *p* = 0.006], and the resting metabolic rate [χ^2^(3) = 21.931, *p* < 0.001] show marked effects during fasting (Figure 3). *Post-hoc* analyses revealed that this was mainly due to a decrease between V0 and V1, sometimes extending to V2, rebounding thereafter again to baseline levels until V3.

#### Glucose Metabolism

Marked changes were seen in plasma glucose [χ^2^(3) = 43.135, *P* < 0.001], HbA1c [χ^2^(3) = 48.921, *P* < 0.001] and fructosamine [χ^2^(3) = 32.056, *P* < 0.001]. During BF, plasma glucose was considerably lower compared with visits before and after the fast. Interestingly, HbA1c was decreased with a temporal delay to the fast, with a smaller decrease between V0 and V1, reaching the minimum only at V2. Here, even values at V3 were lower when compared with baseline values.

Similarly, fructosamine was elevated during and after the fast (V1, V2, and V3 vs. V0), indicating only slow recovery.

#### Chronobiology

**Figure 4:**
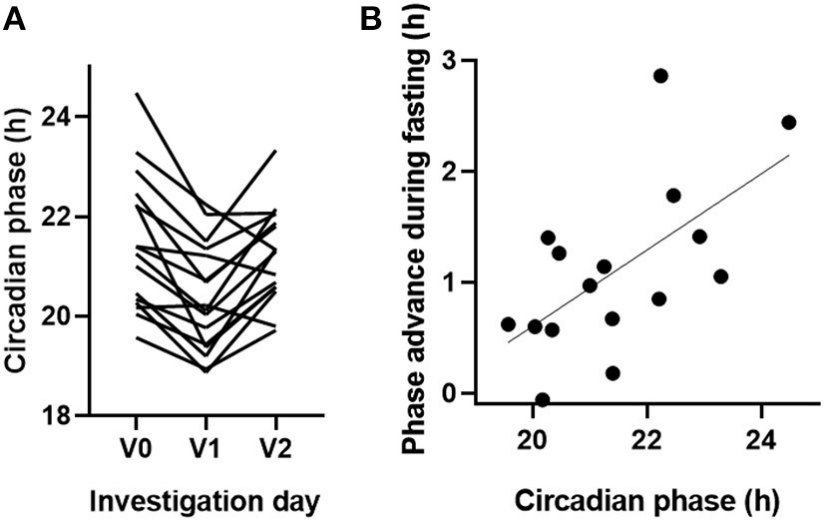
Bahá'í fasting advanced the circadian phase in particular of late chronotypes. **(A)** The circadian phase of 16 study participants was assessed, using the blood monocyte-based BodyTime assay (26) before (V0), during (V1), and 3 weeks after BF (V2). The circadian phase corresponds to the BodyTime-predicted DLMO, which usually occurs about 2 hours before habitual bedtime. **(B)** The extent of advancing the circadian phase during BF correlated with chronotype (i.e., the circadian phase before BF).

The circadian phase (chronotype) of a subject, given by the predicted dim-light melatonin onset (DLMO) assessed, using the blood monocyte-based assay BodyTime (26), varied markedly between the days of investigation [χ^2^(2) = 13.5, *P* = 0.001]. Chronotype during fasting (V1) was notably earlier than in V0 but returned to pre-fasting levels within the next 3 weeks (V2). The extent of advancing the circadian phase correlated distinctly with the initial chronotype, i.e., late chronotypes advanced more than early chronotypes (slope = 0.34, *P* = 0.012, *r* = 0.61, Figure 4). No correlations with other clinical outcomes were found after adjustment.

#### Sex Effects

Repeated-measures ANOVA was used to examine the changes over time in the interaction between sex and visits for BMI, body fat mass, resting metabolic rate, glucose, HbA1c, plasma, and urine osmolality (including 24-hour, diurnal and nocturnal measurements), as well as for the chronobiological data. In this explorative analysis, we could not detect any relevant differences.

## Discussion

This study aimed to generate hypotheses about the health effects and safety of religiously motivated diurnal IDF. Particular emphasis was placed on changes of hydration status, as this aspect is the least scientifically explored until now. Our results showed that hydration status varied slightly between fasting and non-fasting times in this study sample. Although the mean changes in osmolality were within the physiological range, it could, on scrutiny, be observed that some dynamics seem to be present during nighttime, while diurnal samples remain almost unchanged compared with non-fasting samples.

In summary, our data indicate the safety of BF regarding hydration and renal function. Even a slight diluting effect on urine and plasma was observed. Despite a lower resting metabolic rate, the anthropometric indices, as well as glucose metabolism, seemed to profit from BF, without a rebound being witnessed even 3 months later. The phase of circadian rhythms was changed during BF, an effect being caused either by fasting itself or by concomitant behavioral changes.

To our knowledge, this study is the first worldwide to assess physiological and psychological changes during the religious fast of the Bahá'ís. Different methodological approaches were chosen to unravel cross-links between physiological parameters and religious experience and objective and subjective dimensions of this specific fast. This approach was confirmed even in interpreting the laboratory data on the main study focus, which was the hydration status. We showed that, during BF, hydration parameters remained within physiological limits or even indicated a dilution. Both serum and 24-hour-urine osmolality dropped. In the qualitative interviews, some subjects explained they would more consciously drink in the mornings before sunrise (own data, publication pending), which could explain part of the effect. But, as serum osmolality during BF was measured in the afternoon, there might be more effects leading to the slight dilution observed in most subjects. One of these could be activation of the renin–angiotensin–aldosterone system. The RAAS preserves body water resources by retaining sodium and eliminating potassium. According to our data, the latter actually decreased during BF, a finding which is in line with those in Ramadan fasting (31, 32). This would also suit the slight increase in osmolality in the afternoon spot urine samples. In prolonged DF, an increase in RAAS activity has been described (20), as well as an increase in serum osmolality only after 24-hour of DF, while urine volume decreased (15). Our data showed a decrease in both serum and urine osmolality in the 12- and 24-hour samples, while total body water remained unchanged in the bioimpedance analysis. Our findings suggest that, in the population studied, no relevant dehydration occurred during IDF.

Comparing the outcomes of BF on body weight and the metabolic rate with the results from the studies on Ramadan fasting, we see a diverse picture in Ramadan fasting. Some studies report a weight loss, others no change and even others a weight gain during Ramadan fasting (33). Also, it seems that mostly overweight people lose weight in Ramadan, while those with normal weight do not show as much effect (12). As not enough overweight individuals with a BMI ≥ 25 kg/m^2^ were included, this study cannot confirm a similar tendency in BF. A first data analysis of our sub-study at the ECRC indicated an increased adipose tissue lipid mobilization (publication pending). Thus, it could be postulated that BF triggers lipid metabolism, leading to a loss of body fat mass and body weight despite a reduction of the resting metabolic rate.

Furthermore, the observed weight loss and the drop of the resting metabolic rate, alongside the measurement of the activity of the clock genes, show that cyclical changes in energy metabolism influence nutrient utilization and that short-term changes in meal frequency and timing have an effect on chronobiology and energy balance. This is in line with the findings on Ramadan fasting (33).

Previous studies showed that intermittent fasting can affect sleep–wake patterns as well as circadian rhythms in animals and humans. Popular readouts for circadian rhythmicity are repeated measures of body temperature and melatonin levels as well as subjective assessment of chronotype, using questionnaires. Here, a recently developed BodyTime assay was used, in which only one blood sample is needed to objectively assess the phase of the circadian clock of an individual (26). Although the BodyTime assay has not been validated in individuals under specific fasting dietary regimens, it is important to note that, in the study, in which we identified the biomarkers (26), the subjects received hourly isocaloric snacks, whereas, in the subsequent validation study, the participants were free to choose the dietary regimen. At least, when comparing these largely different dietary regimens, we found no difference in accuracy in the circadian phase assessment. The results showed that BF markedly advanced the circadian phase of fasting individuals by more than 1 hour, which is reversed to normal levels 3 weeks after fasting (Figure 4A). In animals, restricting food intake to a few hours within their inactive phase substantially alters circadian rhythms in peripheral tissues, while the master clock in the hypothalamic suprachiasmatic nucleus remains largely unaffected [for a review, see Manoogian and Panda (34)]. Studies investigating how Ramadan fasting affects circadian rhythms revealed conflicting results, since they often cannot discriminate direct effects of fasting on the circadian system from indirect effects of lifestyle changes [for a review, see Qasrawi et al. (35)]. However, it remains unclear whether BF alters the circadian system *per se* or whether the observed phase advance of circadian rhythms is rather due to altered sleeping times and thus altered light exposure, which would phase shift circadian rhythms. A limitation of our study is that we recorded neither light levels nor sleeping time, which would have probably been helpful to discriminate between these hypotheses. However, the fact that we observed larger phase advances for later chronotypes (Figure 4B) supports the second hypothesis, since the participants reported eating and drinking before sunrise (own data, publication pending), which requires relatively earlier wake-up times for late types during BF compared with early types.

A possible bias of the study may originate from the fact that rather healthy, highly educated volunteers participated in this study. This sociodemographic distribution reflects findings from another sample of Bahá'ís in Germany (21). To counteract these biases and to track intraindividual changes during BF, this study was designed as a longitudinal study with a pretest and a longer follow-up than most of the Ramadan studies. As, in Germany, the Bahá'í religion only has ~6,000 followers in more than 100 localities (21), it was a challenge to get an adequate sample size. Future confirmatory studies should be conducted in areas with more Bahá'ís to allow higher participant numbers. Timing of samplings was yet another challenge. During BF, fasting measurements were not possible in the mornings, as breakfast was taken at dawn. Changes seen in physiological parameters during BF may thus include time-of-day effects. To meet this challenge, we included longer-term parameters, for example, 24 h-urine samples for osmolality or HbA1c for serum glucose, as well as follow-up measurements. These show longer-lasting changes, so we can exclude these were only due to time-of-day effects.

Studies with higher participant numbers are needed to validate the hypothesis of urine dilution and weight loss during BF, as well as the improvement of glucose and fat metabolism. Future studies should also focus on exploring the physiological background of these clinical changes and their potential impact on long-term health. It should also be considered to study BF in different cultural contexts, as Bahá'ís fast all over the world. Culturally different approaches to fasting as well as the meal composition could influence the outcomes under discussion.

In summary, this study showed that Bahá'í fasting, although being shorter than other intermittent fasting regimes (5), might have a notable impact on fat and glucose metabolism. Refraining from drinking as well as chronobiological considerations might be crucial in this metabolic shift and should be studied further, especially as this study has not found critical effects on hydration or renal parameters in this kind of intermittent dry fasting.

## Data Availability Statement

The original data of the chronobiological measurements can be found in Supplementary Material. Futher data described in the article will be made available upon request from the corresponding author pending application and approval.

## Ethics Statement

The study protocol was approved by the institutional review board of Charité Universitätsmedizin Berlin (Charitéplatz 1, 10117 Berlin) in January 2018 (ID: EA4/216/17) and was registered with clinical trials (ID: NCT03443739). The participants provided their written informed consent to participate in this study.

## Author Contributions

DK-L conceived and designed the study and had primary responsibility for final content. MB and AMä assisted in designing the part of the metabolic measurements. AK designed the chronobiological part of the study. DK-L, CKl, JS, RR, AMä, MB, BK, and MJ conducted the research. DK-L, CKl, FK, NS, JS, AMä, MB, CKe, RR, AMi, BA, and AK analyzed and interpreted data. DK-L, CKl, FK, AK, AMi, MB, and NS wrote the paper. All the authors have read and approved the final manuscript.

## Conflict of Interest

The authors declare that the research was conducted in the absence of any commercial or financial relationships that could be construed as a potential conflict of interest.

## Publisher's Note

All claims expressed in this article are solely those of the authors and do not necessarily represent those of their affiliated organizations, or those of the publisher, the editors and the reviewers. Any product that may be evaluated in this article, or claim that may be made by its manufacturer, is not guaranteed or endorsed by the publisher.

## Abbreviations

BIA: bioelectrical impedance analysis
BGA: blood gas analysis
BMI: body mass index
BF: Bahá'í fasting
BQ: participants of the large-scale study with questionnaires
CB: participants of chronobiological measurements
DC: Department of Chronobiology
DF: dry fasting
DLMO: dim light melatonin onset
ECRC: experimental and clinical research center
HbA1c: glycosylated hemoglobin
IDF: intermittent dry fasting
IM: Department of Integrative Medicine under the Institute of Social Medicine, Epidemiology and Health Economics
PP: participants of laboratory and anthropometric measurements
V0, V1, V2, and V3: study visits with laboratory examinations
VQ1, VQ2, VQ3, and VQ4: study visits with questionnaires.
