# Supplementary material for: Effects of Daytime Dry Fasting on Hydration, Glucose Metabolism and Circadian Phase: A Prospective Exploratory Cohort Study in Bahá'í Volunteers
Source: Front Nutr. 2021 Jul 29;8:662310. doi: 10.3389/fnut.2021.662310 (PMC8358295; doi:10.3389/fnut.2021.662310)
Supplement: Supplementary file 1 [file Data_Sheet_1.PDF]

Supplementary Table 1: Date and time of blood collection for the BodyTime assay

	T0		T1		T2	
**Participant**	**Date**	**Time**	**Date**	**Time**	**Date**	**Time**
P045	22.02.2018	08:43	15.03.2018	09:52	13.04.2018	09:40
P076	19.02.2018	09:09	20.03.2018	09:05	18.04.2018	08:11
P082	22.02.2018	10:45	20.03.2018	10:10	12.04.2018	10:00
P093	21.02.2018	08:27	16.03.2018	08:45	11.04.2018	08:38
P095	19.02.2018	09:32	20.03.2018	09:40	18.04.2018	09:50
P101	21.02.2018	11:00	14.03.2018	08:43	18.04.2018	08:40
P106	20.02.2018	10:18	14.03.2018	08:10	13.04.2018	08:40
P107	21.02.2018	09:56	16.03.2018	08:20	11.04.2018	09:01
P110	19.02.2018	09:45	19.03.2018	08:25	19.04.2018	08:00
P111	20.02.2018	08:55	20.03.2018	08:40	16.04.2018	08:20
P113	21.02.2018	09:30	16.03.2018	09:58	11.04.2018	08:15
P122	22.02.2018	09:30	15.03.2018	09:42	19.04.2018	08:10
P123	22.02.2018	10:15	20.03.2018	09:55	18.04.2018	10:16
P125	21.02.2018	09:05	14.03.2018	10:05	19.04.2018	09:18
P134	21.02.2018	08:30	19.03.2018	08:05	12.04.2018	08:15
P143	20.02.2018	10:45	14.03.2018	10:20	19.04.2018	08:35
P146	21.02.2018	09:05	19.03.2018	08:10	12.04.2018	08:28
